# RecoverNow: Feasibility of a Mobile Tablet-Based Rehabilitation Intervention to Treat Post-Stroke Communication Deficits in the Acute Care Setting

**DOI:** 10.1371/journal.pone.0167950

**Published:** 2016-12-21

**Authors:** Karen H. Mallet, Rany M. Shamloul, Dale Corbett, Hillel M. Finestone, Simon Hatcher, Jim Lumsden, Franco Momoli, Michel C. F. Shamy, Grant Stotts, Richard H. Swartz, Christine Yang, Dar Dowlatshahi

**Affiliations:** 1 Champlain Regional Stroke Network, Ottawa, Ontario, Canada; 2 The Ottawa Hospital, Ottawa, Ontario, Canada; 3 Ottawa Hospital Research Institute, Ottawa, Ontario, Canada; 4 Brain and Mind Research Institute, University of Ottawa, Ottawa, Ontario, Canada; 5 Canadian Partnership for Stroke Recovery, Ottawa, Ontario, Canada; 6 Élisabeth Bruyère Hospital, Ottawa, Ontario, Canada; 7 Children’s Hospital of Eastern Ontario Research Institute, Ottawa, Ontario, Canada; 8 University of Toronto, Toronto, Ontario, Canada; University of Glasgow, UNITED KINGDOM

## Abstract

**Background:**

Approximately 40% of patients diagnosed with stroke experience some degree of aphasia. With limited health care resources, patients’ access to speech and language therapies is often delayed. We propose using mobile-platform technology to initiate early speech-language therapy in the acute care setting. For this pilot, our objective was to assess the feasibility of a tablet-based speech-language therapy for patients with communication deficits following acute stroke.

**Methods:**

We enrolled consecutive patients admitted with a stroke and communication deficits with NIHSS score ≥1 on the best language and/or dysarthria parameters. We excluded patients with severe comprehension deficits where communication was not possible. Following baseline assessment by a speech-language pathologist (SLP), patients were provided with a mobile tablet programmed with individualized therapy applications based on the assessment, and instructed to use it for at least one hour per day. Our objective was to establish feasibility by measuring recruitment rate, adherence rate, retention rate, protocol deviations and acceptability.

**Results:**

Over 6 months, 143 patients were admitted with a new diagnosis of stroke: 73 had communication deficits, 44 met inclusion criteria, and 30 were enrolled into RecoverNow (median age 62, 26.6% female) for a recruitment rate of 68% of eligible participants. Participants received mobile tablets at a mean 6.8 days from admission [SEM 1.6], and used them for a mean 149.8 minutes/day [SEM 19.1]. In-hospital retention rate was 97%, and 96% of patients scored the mobile tablet-based communication therapy as at least moderately convenient 3/5 or better with 5/5 being most “convenient”.

**Conclusions:**

Individualized speech-language therapy delivered by mobile tablet technology is feasible in acute care.

## Introduction

Communication disorders are common after stroke. Approximately 40% of patients diagnosed with a stroke will have aphasia [1]; intensive speech and language therapy is recommended in order to maximize the chances of recovery [2–3]. Initiating aphasia therapy early after stroke improves communication outcomes [4, 5], and these improvements can persist for at least 12 months [6]. A Cochrane Review [7] identified significant improvements to functional communication, reading comprehension and expressive language function when patients with stroke received speech and language therapy compared to those who did not.

Speech and language rehabilitation should ideally start at the time of acute hospitalization [8]. Unfortunately, in Canada, access to speech and language therapy is often delayed due to unavailability of timely rehabilitation services: in 2014, only 16% of Canadians with stroke were able to access in-patient rehabilitation, and of those, only 50% accessed rehabilitation centers within two weeks of their stroke [9]. During these first two weeks of acute care, patients spend over 60% of their time inactive and alone [10]. Rehabilitation experts in acute care centres can use this “down time” as an opportunity to leverage existing mobile platform technologies programmed with speech and language therapy applications [11, 12, 13] and deliver timely interventions.

Ongoing studies are testing the feasibility of using mobile devices to deliver stroke rehabilitation [14, 15], with preliminary studies supporting their role in outpatient aphasia therapy [12, 16, 17]. A recent qualitative study suggested stroke survivors found such interventions helpful and acceptable [18]. While mobile devices have been introduced in the acute care setting to promote patient education and engagement [19, 20], there have been no studies using mobile platform technologies for communication therapy, specifically within the first 14 days following stroke.

There are potential barriers to introducing mobile platform technologies in the acute stroke setting, including patient-related factors such as the severity of the stroke and resulting deficits (e.g. vision, hemiparesis, neglect), intervention-related barriers such as complexity of the hardware or software applications, and system-related barriers such as reliable and secure wireless access in the hospital. Furthermore, Speech-Language Pathologist (SLP) assessments are crucial to identify patients who may best benefit from speech and language therapy interventions. Yet SLPs are not consistently consulted for assessments at the time of patient admission; a 5-year Canadian hospital audit revealed that over a third of patients discharged with aphasia did not receive SLP assessment or treatment during acute care [21].

Despite these potential barriers, we hypothesized it would be possible to initiate communication therapy using mobile platform technology in the acute care setting. We sought to test the feasibility of using mobile tablets in the acute care setting to deliver speech and language therapy to patients admitted with acute stroke. Our primary feasibility objective was to assess recruitment rate, adherence rate, retention rate, protocol deviations, and acceptability. Our secondary objective was to describe any barriers encountered at the intervention level, patient level, and systemic level.

## Materials and Methods

### Study design

This study was a prospective observational cohort design approved by the Ottawa Health Science Network Research Ethics Board. Patients were enrolled based on a waiver of consent process (no written or verbal consent was required) based on the speech-language therapy intervention fitting within the College of Audiologists and Speech-Language Pathologists of Ontario clinical standard-of-care [22], and consistent with the Tri-counsel Policy Statement 2 for Ethical Conduct for Research Involving Humans (article 3.7). The study was conducted according to the principles expressed in the Declaration of Helsinki.

### Participants

We enrolled all consecutive patients admitted to the Neurology inpatient service of The Ottawa Hospital (Ontario, Canada) with a diagnosis of stroke and, 1) mild to moderate communication deficits in any of the following domains: speech, language and/or cognitive communication, and/or 2) Scoring ≥ 1 on the Best Language and/or Dysarthria parameters of the National Institute of Health Stroke Score (NIHSS) [23]. Exclusion criteria were, 1) Pre-existing speech, language or cognitive disorders (such as dementia), 2) Severe debilitating disease(s) that, in the opinion of the investigator, would preclude them from being able to perform the required tasks of the study on the tablet (ex: end-stage malignancy, ALS), 3) Severe comprehension deficits (unable to follow simple one-step commands and/or unable to respond to yes/no questions reliably), and 4) Inability to perform tasks in English.

### Procedures

Patients were evaluated neurologically and for any communication problems using standard neurological and communication assessments. Patients with any speech, language or cognitive-communication deficit received a consultation from an experienced SLP during their acute inpatient stay. The initial face-to-face contact with the SLP was consistent with current SLP standard of care for patients with communication deficits (i.e. consent for assessment, an initial assessment, followed by the provision of counseling/education/exercises with regards to their communication deficits). This standard of care SLP assessment took between 30 and 60 minutes and involved the use of commonly used assessment tools at the discretion of the therapist, including: Western Aphasia Battery–Revised Bedside (Bedside WAB) [24], Western Aphasia Battery—Revised (WAB-R) [25], Boston Diagnostic Aphasia Examination– 3^rd^ Ed. (BDAE-3) [26], Boston Naming Test– 2^nd^ Ed. (BNT) [27], Ross Information Processing Assessment–Second Edition (RIPA-2) [28], Oral-Motor and Speech Assessments from The Source for Dysarthria– 2^nd^ Ed., 2010 [29]. If a patient satisfied all inclusion criteria, they were offered a mobile tablet by the SLP and enrolled in the study by the research coordinator. Once a patient was enrolled in the study, we collected basic demographic data in addition to information on education, computer knowledge and previous mobile tablet usage.

Each patient enrolled in the study was provided with an iPad Air (Apple ®, Cupertino, California, United States), which was housed in a carrying case. Each iPad was loaded with commercially available applications deemed appropriate for impairment-based therapy selected by the SLP to target the communication deficits identified by the communication assessment (Table 1). The SLP spent a maximum of 15 minutes training the patient on the mobile tablet as an introductory session, with a follow-up (maximum 10 minutes) completed on the next business day to answer any questions that arose. The introductory training was provided to the patient and family member, friend or caregiver (when available) on the use of the iPad. This included explicit explanations on how to open the iPad, how to turn it on, how to login (password was written on the inside of the case for reference), how to access the apps, how to perform the tasks within the selected apps, and how to get out of the apps. Once the explicit teaching was done, patients and/or the family/friend/caregiver had to demonstrate their ability to do these tasks. At the follow-up, questions were answered and any problem solving was completed with the patient/family member. Furthermore, our Research Coordinator visited the patient daily (business days) to gather information on usage time as well as help with any technical issues, including device recharging. As our institution only utilizes SLP for initial assessments and recommendations for post-acute care rehabilitation needs, there were no additional speech-language therapies provided beyond the mobile tablet applications.

**Table 1:** Commercially available applications and their use.

Applications	Target Patient Group
Constant Therapy (Newton, MA, USA)	Mild to moderate language and/or cognitive-communication deficits.
Tactus Therapy Solutions Ltd. (Vancouver, BC, Canada)Language Therapy 4-in-1 Therapy Toolkit	More severe language deficits.
Tactus Therapy Solutions Ltd.(Vancouver, BC, Canada)Question Therapy 2-in-1: Asking and Answering Questions	Milder language and/or cognitive-communication deficits.
Tactus Therapy Solutions Ltd. (Vancouver, BC, Canada)Category Therapy	Milder language and/or cognitive-communication deficits.
Tactus Therapy Solutions Ltd.(Vancouver, BC, Canada)Conversation Therapy Gets People Talking	Milder language and/or cognitive-communication deficits.
Lingraphica SmallTalk Oral Motor Exercises (Princeton, NJ, United States	Motor speech deficits
Multimedia Speech Pathology Speech Soundson Cue for iPad (Coolangatta, QLD, Australia)	Motor speech deficits
Jay Bacal apps (Mahopac, NY, USA) with the modules Search 4 It, Chain Of Thought,Morphos, Get + Together and This Is To That	Milder language and cognitive-communication deficits.

The Canadian Stroke Best Practice Recommendations for Rehabilitation indicate “patients should receive a minimum of three hours of task-specific therapy, five days a week delivered by the interprofessional team” [30]. As such, we recommended that the patient spend at least one hour of therapy per day on the tablet throughout their stay in the acute care setting. Patients had access to Wi-Fi, but access to non-study applications was restricted; we used the iOS “restrictions” and our hospital IT security office independently confirmed that only study apps could be accessed, with the exception of preloaded Apple apps that cannot be restricted (Clock, Contacts, Calendar, Tips, Reminders, Maps, Notes and Settings).

### Outcome measures and data analysis

Our primary outcome was feasibility, measured as recruitment rate (number of patients enrolled/total patients meeting study criteria), adherence to protocol (interacting with the mobile device for an average of one hour/day), retention rate (number of patients continuing to use the mobile tablet until the time of discharge), protocol deviations, and acceptability using a five-point Likert scale. Protocol adherence was measured using the iOS usage data; a study coordinator would check the device usage each day. Protocol deviations were defined as any unplanned or unforeseen change from the approved protocol. They were monitored and recorded by the assigned research coordinator and regularly reviewed by the principal investigator to determine its overall impact on the study, as required by our Research Ethics Board. The Likert scale had two anchors, an “extremely” positive option on one end and “not at all” negative option on the other end. The secondary objective was to describe any barriers encountered at the intervention level, patient level, and systemic level. Descriptive statistics were provided and expressed as medians with interquartile ranges (IQR).

## Results

### Recruitment

**Fig 1:**
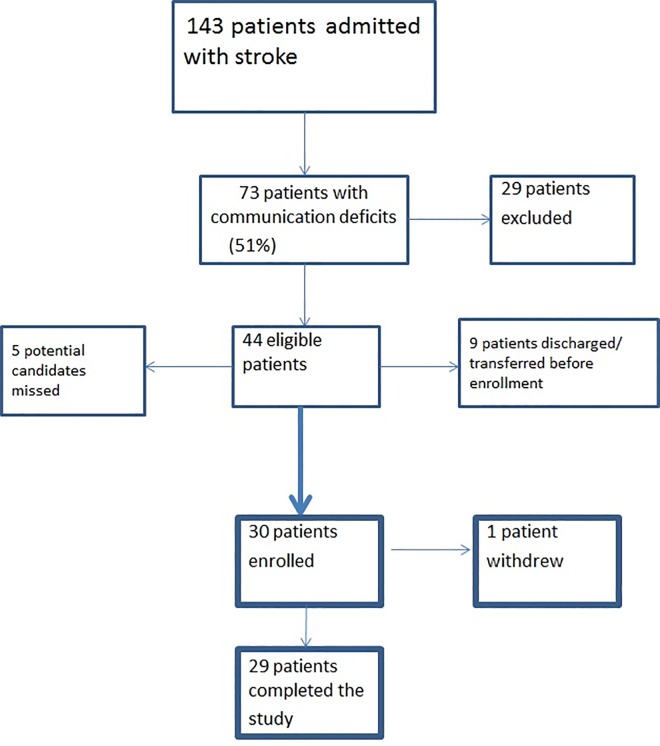
Flowchart of patients enrolled in the study.

Over the period of six months (December 2014-June 2015) 143 patients were admitted into our institution with a diagnosis of stroke. Of the 143 patients, 73/143 (51%) had communication deficits. Twenty-nine were excluded based on our criteria (11 with severe co-morbidities, two had severe communication deficits, three were non-English speaking and 13 were palliative). Of the remaining eligible 44/143, nine patients were discharged or transferred to another facility prior to enrollment and five potential candidates were missed (Fig 1). Therefore, 30/44 eligible (68% recruitment rate) were enrolled in the study (Table 2).

**Table 2:** Baseline demographics of enrolled patients.

Age in years, range, median (IQR)	35–92, 62 (25)
Sex	73.4% male
Type of stroke	
• Left MCA	13/30 (43.3%)
• Right MCA	5/30 (16.7%)
• Others	12/30 (40%)
Vascular history	
• Hypertension	12/30 (40%)
• Diabetes Mellitus	6/30 (20%)
• Dyslipidemia	5/30 (17%)
• Coronary Artery Disease	3/30 (10%)
• Smoking	3/30 (10%)
Admission AlphaFIM^®^	66 (47–90)
Level of Education	
• High School (no diploma)	5/30 (16.7%)
• High School Diploma	3/30 (10%)
• College Degree	10/30 (33.3%)
• University Degree	7/30 (23.3%)
• Postgraduate Degree	4/30 (13.3%)
• No formal education	1/30 (3.3%)
Computer Knowledge (self-declared)	
• None	3/30 (10%)
• Beginner	5/30 (16.7%)
• Average	17/30 (56.7%)
• Advanced	5/30 (16.7%)
Previous iPad usage	
• Yes	21/30 (70%)
• No	9/30 (30%)
Number of days from admission to study enrolment, Median (IQR)	5 (6)
Duration (in minutes) of mobile tablet use per day	Mean (SEM) = 149.8(19.1)Median (IQR) = 128.8 (90–187)

### Retention, Adherence, Protocol Deviations and Acceptability

In-hospital retention rate was 97% (29/30). One patient declined to continue because he found working with mobile technology challenging. The median (IQR) length of stay in acute care was 19.5 days (10.25–24.75), and the median (IQR) time with the tablet was 10 days (5–14.75).

**Fig 2:**
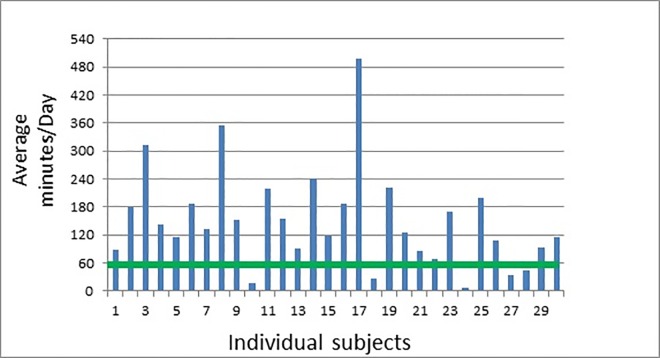
Individual Adherence Data. The blue vertical bars indicate the average number of minutes per day spent interacting with the mobile tablet for each subject. The green horizontal bar is set at the target 60 minutes per day recommended by the SLP.

The average time spent each day with the mobile tablet is shown in Fig 2; patients interacted with the mobile tablet for an average of 149.8 minutes/day, and 25/30 (83%) were on average able to complete the recommended one hour/day. The majority of the activity (86.3%) occurred between noon and 8pm. Patients 3, 8 and 17 (Fig 2) spent on average greater than 5 hours per day on the mobile device. Patient 3 was a 60-year old male accountant with mild receptive aphasia and moderate dysarthria, and average computer experience. Patient 8 was a 55-year old male engineer with moderate to severe expressive language deficits and above average computer experience. Both were extremely motivated to improve their language function and expressed the desire to work on the tablet. Patient 17 was a 61-year old male mechanic with mild to moderate receptive and expressive language deficits. He had never previously used a mobile tablet and described himself as a “beginner” with computers. He used the tablet whenever possible, often in different areas of the stroke ward. Conversely, patient 10 was a 73-year-old retired grocery store worker with mild flaccid dysarthria who used the tablet minimally for oral motor exercises and speech sounds. Similarly, patient 18 was a 57-year-old car salesman with moderate receptive and expressive language deficits who found it challenging to use the tablet; subsequent neuropsychological assessment revealed cognitive impairment. Patient 24 was a 72 year old retired newspaper receiver with mild-moderate receptive aphasia and moderate expressive aphasia. He used the tablet for only 7 minutes then discontinued and withdrew from the study, stating he felt too fatigued to participate.

Protocol deviations included one charger being lost, and one patient left the hospital with the mobile tablet and charger that were later retrieved by the study team. The majority of patients (23/25, 92%) rated the mobile tablet as 3/5 (moderately convenient) or better on the iPad Convenience Survey (Table 3).

**Table 3:** Responses to iPad convenience survey (n = 25).

* *	Extremely easy/ clear/ convenient	Very easy/ clear/ convenient	Moderately easy/ clear/ convenient	Slightly easy/ clear/ convenient	Not at all
1- How easy was it to hold the iPAD?	3 (12%)	10 (40%)	11 (44%)	0	1 (4%)
2- How clear were the instructions to use the apps on the iPAD?	3 (12%)	7 (28%)	12 (48%)	1 (4%)	2 (8%)
3- How easy was it to use the apps on the iPAD?	3 (12%)	11 (44%)	6 (24%)	5 (20%)	0
4 How easy was it to send/transfer your responses after completion of each app?*	0	2 (50%)	2 (50%)	0	0
5- Overall, how convenient was it to use the iPAD in your therapy?	1 (4%)	15 (60%)	7 (28%)	2 (8%)	0

* Four patients used an app that required them to submit online responses to the SLP.

### Barriers

We encountered three levels of barriers during the execution phase of this study: patient, intervention and system barriers. Patient-related barriers included one patient reporting a feeling of embarrassment talking to the mobile tablet in public, which was resolved with the use of a headset. Some patients required additional time and family support to understand the intervention. One intervention-related barrier was difficulty in opening mobile tablet cases due to deficits with fine motor control; a substitute case was found which resolved this issue (Belkin Stripe iPad Mini 1/2/3 Folio Case). A second intervention barrier was the need to disinfect the mobile tablet, the case, and the charger between users to satisfy hospital Infection Control requirements. This was achieved by wiping the iPad and the iPad case with hospital-approved alcohol or hydrogen peroxide wipes once they were returned to the study coordinator at the time of discharge. System-related barriers included initially inconsistent Wi-Fi access, which was resolved using a dedicated Information Technology (IT) supported server. There were also concerns from IT security, which required an independent assessment to ensure the privacy of patients and security of the Hospital wireless network and servers. Lastly, when patients presented with communication deficits at the time of admission, early consultation to SLP services was not consistently done, which contributed to the five missed patients who were otherwise eligible (Fig 1).

## Discussion

Despite the evidence and recommendations for early rehabilitation [2,3,4,30], patients with stroke are often inactive and alone during their acute care hospitalization: in the 14-day period following a stroke, patients spend over 50% of their time resting in bed, and 60% of their time alone [10]. This acute care “downtime” provides an opportunity for targeted rehabilitation interventions using mobile platform technologies that can be utilized in wardrooms without supervision, or additional health care personnel. Arguably, communication therapies are particularly well suited for this paradigm, as they can be administered in bed with minimal risk of injury and/or falls. The present study evaluated the feasibility of providing mobile tablet-based communication therapy to patients in the acute care setting immediately after having sustained a stroke. In keeping with Canadian Best Practice Recommendations for Stroke Care [30], patients were instructed to interact with the mobile device for at least one hour per day during their downtime; on average, patients more than doubled this recommendation (although we cannot be certain they spent this time using only the therapy applications). We found that it was feasible for patients to use mobile tablets for communication therapy while in the acute care setting immediately following a stroke. Indeed, many patients expressed a desire to have a more active role in their therapy and exceeded the recommended one-hour minimum. We therefore propose it is feasible to launch a clinical trial assessing the efficacy of mobile-tablet based communication therapy in the acute care setting, and we provide basic recruitment, retention and adherence data to inform its design.

While early and intensive communication therapy is recommended for patients following a stroke [2–4], there is limited evidence to support the use of mobile technology in this regard. Mobile tablets have the potential for maintaining and augmenting communication abilities in the home setting [17], with published reports supporting their ease of use [18]. Pilot studies suggest tablet-based interventions may complement language and cognitive therapy in patients with post-stroke communication disorders [17,31,32]. However, these early studies applied mobile device interventions between one month and 178 months following stroke; to our knowledge, our study is the first to specifically target the 14-day early acute care hospital stay.

While half of our patients started communication therapy within the first week, therapy should ideally start sooner to optimize the critical stroke recovery window [33–37]. A few factors may have led to a 6.8 day median enrollment: SLP was not always consulted immediately at the time of admission, admission over weekends or holidays led to assessment delays, and subtle communication deficits may have initially been missed due to a focus on gross motor and gait deficits. Furthermore, five patients meeting inclusion criteria were missed because of early discharge suggesting that more consistent consultations to SLP could have improved the recruitment rate.

The barriers encountered in our study can be classified into three levels: patient, intervention and system. Patient barriers included a preference to use headphones in certain patients, and the need for training and support. The training time was minimal, consisting of a maximum 15 minute introductory session, and a 10 minute follow-up session. Support was equally minimal, and provided by a Research Coordinator who checked in daily to ensure the charging of the device battery, and to record usage data. The intervention barriers encountered included: 1) difficulties in opening the iPad case, and 2) Infection Control highlighted the need to disinfect devices and cases between users. The default cases that came with iPads were not malleable, and opened/closed by snap-ons. Furthermore, it was difficult to tilt iPads to an upright position. After reviewing several alternatives, we found suitable malleable cases (Belkin Stripe iPad Mini 1/2/3 Folio Case) that allowed easier opening/closing via their magnetic strap; these also had better maneuverability. We found facilitating interactions with the iPad by obtaining an appropriate case was important in this stroke population, given the prevalence of motor deficits in addition to communication problems. The study coordinator disinfected devices between users with hospital-approved alcohol or hydrogen peroxide wipes. This process was reviewed and accepted by our hospital Infection Control team.

While our relatively small sample size does not provide a comprehensive assessment of potential barriers to implement a mobile tablet acute rehabilitation therapy program, it does provide some basic information on issues that must be addressed in designing further prospective studies. The greatest system challenge was to ensure that devices were compliant with the high security and privacy standards required by acute care hospitals. While patients and their caregivers are unlikely to violate privacy and security protocols, there is always a potential for unauthorized individuals to gain access to the devices and the Hospital wireless system. This systemic barrier is best overcome by working closely with the institutional Information Systems team during the planning and deployment phase, and at the time of any software/hardware modification.

While our study demonstrates the feasibility of introducing mobile tablets in the acute care setting for the purpose of rehabilitation for post-stroke communication deficits, there are important limitations to the design. First, we did not assess the long-term retention and adherence to the treatment protocol, as the study was limited to acute inpatient hospital stay. It is not known if patients will continue to use the devices for a prescribed time-point following discharge, and whether they will return for study follow-up with minimal attrition. Second, we have not addressed the feasibility of continuing in a mobile-platform therapy study across health-care transition points. Based on our experience, we would expect additional IT security concerns when transitioning a patient with a mobile tablet between health-care centres; this will require cooperation between IT departments to ensure a seamless transition with minimal disruption to the treatment protocol. Third, we cannot exclude that our usage data used to calculate adherence included the use of non-study apps preloaded on the device (Clock, Contacts, Calendar, Tips, Reminders, Maps, Notes and Settings). Finally, our study did not address the efficacy of the intervention and we did not collect performance data, as our primary objective was one of feasibility. Without this performance data, there was no mechanism to quantify the amount of time spent actively engaging with the tablet-therapy as opposed to superficially scanning applications, nor was there any mechanism to quantify the time spent on each individual application. Further, there is no data to help identify responders versus non-responders. To address these points, additional studies are required to establish the efficacy of early mobile-platform based stroke rehabilitation therapy.

## Conclusion

Mobile tablet technologies may facilitate rehabilitation interventions during the early hospitalization period following acute stroke. Our study suggests mobile tablets are a feasible method to deliver individualized communication therapy in the acute care setting, and it is reasonable to proceed to a clinical trial. We provide recruitment, retention and adherence rates to inform the design of a randomized controlled trial assessing the efficacy of mobile tablet-based communication therapy interventions in the acute post stroke period.
